# A Whiff of Garlic, a Turn of Fate: Recognizing Organophosphorus Poisoning Amid Diagnostic Uncertainty: A Case Report

**DOI:** 10.1002/ccr3.71722

**Published:** 2025-12-29

**Authors:** Sujan Tamang, Bishwo Ram Amatya, Sulav Neupane, Sunischit Chandra Neupane, Chiranjivi Panta, Anusmriti Pal, Dipesh Shrestha

**Affiliations:** ^1^ Nepalese Army Institute of Health Sciences Kathmandu Nepal; ^2^ Department of Anesthesia Shree Birendra Hospital Kathmandu Nepal; ^3^ Department of Internal Medicine Shree Birendra Hospital Kathmandu Nepal; ^4^ Critical Care Unit, Shree Birendra Hospital Kathmandu Nepal

**Keywords:** diagnostic challenge, organophosphate‐induced delayed neuropathy, organophosphorus poisoning, toxicology, ventilator associated pneumonia

## Abstract

OP compounds, commonly used as pesticides, are readily available over the counter and frequently implicated in intentional poisoning. We report a case of a 43‐year‐old male with OP poisoning initially misdiagnosed as alcohol intoxication. He was intubated in the emergency department on arrival due to a low Glasgow Coma Scale (GCS) of 6/15 and then admitted to the Intensive Care Unit (ICU) where he developed 5 episodes of generalized tonic clonic seizure (GTCS) and ventilator‐associated pneumonia (VAP), progressing to severe acute respiratory distress syndrome (ARDS). The patient required reintubation after initial extubation as he developed an episode of GTCS with sudden loss of consciousness and respiratory arrest. Later, he developed a left foot drop with delayed conduction on the peroneal nerve, suggestive of organophosphate‐induced delayed neuropathy (OPIDN). This case highlights the diagnostic challenges of OP poisoning and potential complications associated with its management.

## Key Clinical Message

Organophosphorus (OP) poisoning is frequently misdiagnosed, especially when exposure history is unclear, and may mimic conditions like alcohol intoxication. Simple bedside clues, including subtle signs of garlic‐like odor and diagnostic tools like atropine challenge, are vital. Early recognition and prompt intervention are crucial in reducing morbidity and mortality.

**Abbreviations**
2‐PAM: 2‐Pralidoxime
ARDS: Acute Respiratory Distress Syndrome
ED: Emergency Department
GCS: Glasgow Coma Scale
GTCS: Generalized Tonic Clonic Seizure
ICU: Intensive Care Unit
NG: Nasogastric
OP: Organophosphorus
OPIDN: Organophosphate‐induced delayed neuropathy
OR: Odds Ratio
VAP: Ventilator Associated Pneumonia

## 1 Introduction

OP poisoning is common in developing countries like Nepal since it is a cheap and effective means of pest control. The main source of exposure is absorption through the mucous membrane, skin, conjunctiva, respiration, and gastrointestinal tract as a result of intentional or accidental ingestion. OP exposure affects about 3 million people worldwide annually, with a mortality rate of 10% [1].

OPs irreversibly inhibit acetylcholinesterase, resulting in the accumulation of acetylcholine at muscarinic and nicotinic sites. This may present as an acute cholinergic crisis, intermediate syndrome, or OPIDN [2]. The bond between OP and acetylcholinesterase becomes irreversible over time, and administration of 2‐pralidoxime (2‐PAM) is crucial [3]. Key muscarinic effects include excessive sweating, salivation, lacrimation, bronchospasm, vomiting, abdominal cramps, miosis, bradycardia, hypotension, and incontinence. Nicotinic effects manifest as muscle fasciculations, weakness, and paralysis. Central effects range from headache and confusion to seizures and coma, with respiratory failure being the leading cause of death. Motor impairment, psychosis, depression, memory, and cognitive flexibility are chronic neuropsychiatric issues that follow acute exposures [4].

Standard management consists of prompt resuscitation, pharmacologic blockade of muscarinic receptors using atropine, reactivation of organophosphate‐inhibited acetylcholinesterase with pralidoxime, and supportive ventilation for patients with respiratory failure [5]. The role of atropine in treating acute organophosphate poisoning is well established. A systematic review and meta‐analysis of randomized trials reported that pralidoxime does not provide significant clinical benefit, consistent with other published studies [6].

The incidence of VAP among patients on invasive ventilation for over two days ranges from 5% to 40%, though the reported rates differ widely across countries, ICU settings, and diagnostic criteria [7].

## 2 Case Presentation

A 43‐year‐old male, retired army personnel, was presented to the emergency department (ED) by relatives after being found unresponsive for 4 h. History suggests that the patient had consumed alcohol over a family dispute and was left outside the house overnight. Later, he was discovered grunting, with urinary incontinence and cold extremities, for which he was rushed to the hospital. Initial vital parameters in the ED revealed tachycardia (126 beats per minute), hypotension (90/60 mmHg), hypothermia (96°F), tachypnea (29 breaths per minute), and SpO2 of 93%. GCS was 6/15 (E2V2M2), and systemic examination revealed bilateral basal crepitations. Due to the poor GCS and risk of aspiration, the patient was intubated upon arrival.

## 3 Initial Investigations

Initial findings on laboratory parameters were suggestive of mixed acidosis with pH of 6.881 (7.35–7.45), pCO2 of 54 mmHg (35–45 mmHg), HCO3 of 8.9 mmol/L (22–26 mmol/L), anion gap of 30 mmol/L (8–12 mmol/L) and lactate of 8.4 mmol/L (0.5–2.2 mmol/L), hyperbilirubinemia with total serum bilirubin of 2.83 mg/dL (< 1.2 mg/dL) and acute kidney injury (AKI) with creatinine level of 2.8 mg/dL (0.7–1.3 mg/dL).

## 4 Differential Diagnosis, Investigations, and Treatment

Based upon history, clinical findings, and laboratory investigations, a provisional diagnosis of ethanol intoxication with severe metabolic acidosis and aspiration pneumonia was made. The patient was immediately transferred to the ICU, and supportive measures, including fluid resuscitation, vasopressors, broad‐spectrum antibiotics, metabolic acidosis correction, and seizure management, were commenced and kept under mechanical ventilation. The patient experienced 5 episodes of GTCS requiring Inj. Midazolam boluses.

On day 2 of ICU stay, the condition of the patient deteriorated with persistent acidosis, fever, and hyperglycemia. A strong garlic‐like odor was detected by the bedside nurse from the nasogastric (NG) free drain. This raised suspicion of OP poisoning. Owing to this, an atropine challenge was initiated. This resulted in an increase in heart rate. Given the serum cholinesterase level of < 400 U/L (4620 U/L–11,500 U/L), the diagnosis of OP poisoning was established, and atropine, along with pralidoxime infusion, was initiated subsequently.

On day 4, the patient gradually improved clinically with atropine therapy; however, as a result of prolonged ICU stay of more than 3 days and mechanical ventilation, VAP developed with isolation of *Acinetobacter baumanni* on tracheal aspirate for which he was treated with Polymyxin B and Minocycline. The patient developed progressive hypoxemic respiratory failure secondary to ARDS requiring a session of proning for nearly 18 h.

On day 5 of ICU admission, the patient passed a spontaneous breathing trial and was extubated. A few hours later, the patient was reintubated because of the recurrence of GTCS and respiratory arrest, and extubated 3 days later. After extubation, a detailed neurological examination was performed, which revealed a left foot drop. Nerve conduction study showed delayed conduction on the left common peroneal nerve, which confirmed the foot drop suggestive of OPIDN. The patient was kept in the ICU for a total of 19 days, following which he was shifted to the general ward. In the ward, he was kept under observation and supportive care, including physiotherapy was given over the next 2 weeks. Neurology consultation was sought for the evaluation of foot drop. He was advised to follow up in the neurology outpatient department; however, despite several attempts of mobile calls, he was lost to follow‐up.

## 5 Discussion

OP poisoning remains a significant threat to health, particularly for those in developing countries where these substances are used as pesticides and are easily available. It can be difficult to diagnose a case of OP poisoning when a patient presents with altered sensorium with no clear and reliable history from family members or friends.

Our case also highlights a similar diagnostic dilemma, which arose due to the lack of a clear clinical history, combined with clinical similarities to alcohol intoxication, such as impaired consciousness, hypoventilation, and low body temperature. Uniquely, it documents a rarely reported progression of OP poisoning, marked by initial clinical improvement followed by delayed complications including ARDS, seizure‐related reintubation, and OPIDN.

In resource‐constrained settings of South Asia, the precise history of poisoning is often vague or intentionally concealed, either due to social stigma or potential legal consequences. Pandit et al. [8] highlighted the challenges in diagnosing OP poisoning when a patient presents with symptoms that are not typical and a history that is unclear. In many cases, including ours, co‐ingestion of alcohol with pesticides confounds the clinical assessment and contributes to diagnostic uncertainty. A recent meta‐analysis by Eddleston et al. (2022) [9] suggested that co‐ingestion of alcohol worsens the clinical outcome in OP poisoning. The odds of death were increased to nearly fivefold with an odds ratio (OR) of 4.9, and the odds of requiring intubation were increased by eightfold (OR = 8.0). This also highlights the need for a high index of suspicion when such co‐exposure of OP compounds and alcohol is possible [10].

The bedside clinical assessment was critical in identifying an important sign of OP poisoning, such as a garlic‐like odor coming from the nasogastric tube as in our case. Although the exact compound could not be confirmed, it is likely that the patient ingested a commonly available pesticide such as malathion or chlorpyrifos, widely used in Nepal. The garlic‐like odor from the nasogastric drainage may indicate a sulfur‐containing organophosphate, providing a useful clinical clue to guide diagnosis in resource‐limited settings. The diagnosis was reconsidered in view of the persistent unexplained metabolic acidosis. The atropine challenge test, along with copious secretions and auscultation findings of bilateral crackles, helped in confirming cholinergic toxicity, particularly in a resource‐limited setting where access to serum cholinesterase measurement is limited or delayed [11]. In relation to a retrospective study, clinicians often rely on supportive management as well as bedside assessments whenever history is not available or unclear, which could result in significant impacts on patient outcomes [12].

Prolonged ICU stays and mechanical ventilation are often necessary in the case of severe OP poisoning, specifically for patients experiencing complications that include seizures, ARDS, and intermediate syndrome. Our patient developed VAP, a common complication that has been reported in 5% to 40% of patients requiring mechanical ventilation [13]. The length of the ICU stay had been substantially longer in our case due to the development of VAP, ARDS, and seizure episodes. According to Patil et al. (2016) and a case series from Nepal [14], a longer duration of mechanical ventilation in the case of OP poisoning, for more than seven days, has been linked to a substantial rise in both morbidity and mortality, largely due to complications including ventilator‐associated pneumonia, sepsis, and long‐lasting neuromuscular weakness [15].

OPIDN is an established delayed complication of OP poisoning that has been linked to the inhibition of neuropathy target esterase, a process that initiates axonal degeneration predominantly targeting motor neurons [16]. OPIDN usually appears one to three weeks after the initial exposure, like a left foot drop seen in the patient. A prospective study conducted in North India showed that 34.8% of OP poisoning cases developed OPIDN [17]. In our study, a nerve conduction study was done that helped us in confirming the condition and also aided us in differentiating it from critical illness neuropathy. He was advised neurologic follow‐up, but was lost to follow‐up after discharge, a challenge frequently encountered in OPIDN cases. This highlights the challenges in ensuring continuity of care among survivors of organophosphate poisoning, where late‐onset complications may go unmonitored or untreated [17].

## 6 Conclusion

This case highlights the diagnostic difficulties associated with OP poisoning, particularly in contexts where the exposure history is either unavailable or intentionally concealed. The initial misdiagnosis as alcohol intoxication delayed the administration of specific antidote therapy and emphasized the importance of careful bedside assessment. Subtle clinical cues, such as the detection of a garlic‐like odor from nasogastric drainage, and the application of a straightforward atropine challenge, were instrumental in guiding the correct diagnosis. Furthermore, this case illustrates the significant complications that may arise during intensive care, including ventilator‐associated pneumonia, acute respiratory distress syndrome, recurrent seizures, and delayed peripheral neuropathy linked to OP exposure. Prompt clinical suspicion, early initiation of appropriate treatment, and proactive management of critical care complications are essential to improving outcomes. The case also underlines the pressing need for clinical vigilance and standardized protocols, particularly in low‐resource settings where access to toxicological diagnostics may be limited.

## Author Contributions

**Sujan Tamang:** conceptualization, methodology, project administration, resources, writing – original draft, writing – review and editing. **Bishwo Ram Amatya:** conceptualization, resources, supervision, writing – review, and editing. **Sulav Neupane:** conceptualization, formal analysis, project administration, resources, writing – review and editing. **Sunischit Chandra Neupane:** conceptualization, methodology, project administration, resources, writing – review and editing. **Chiranjivi Panta:** investigation, supervision. **Anusmriti Pal:** investigation, supervision. **Dipesh Shrestha:** investigation, validation, writing – review, and editing.

## Funding

The authors have nothing to report.

## Ethics Statement

The authors are accountable for all aspects of the work in ensuring that questions related to the accuracy or integrity of any part of the work are appropriately investigated and resolved. All procedures performed in this study were in accordance with the ethical standards of the institutional and national research committee(s) and with the Helsinki Declaration (as revised in 2013).

## Consent

Written informed consent was obtained from the patient for the publication of this case report.

## Conflicts of Interest

The authors declare no conflicts of interest.

## Data Availability

The authors have nothing to report.
